# Supplementary material for: Whole Genome Sequencing of the Blue Tilapia (Oreochromis aureus) Provides a Valuable Genetic Resource for Biomedical Research on Tilapias
Source: Mar Drugs. 2019 Jun 28;17(7):386. doi: 10.3390/md17070386 (PMC6669741; doi:10.3390/md17070386)
Supplement: Supplementary file 1 [file marinedrugs-17-00386-s001.zip › Supplementary Information/Figure S1.pdf]

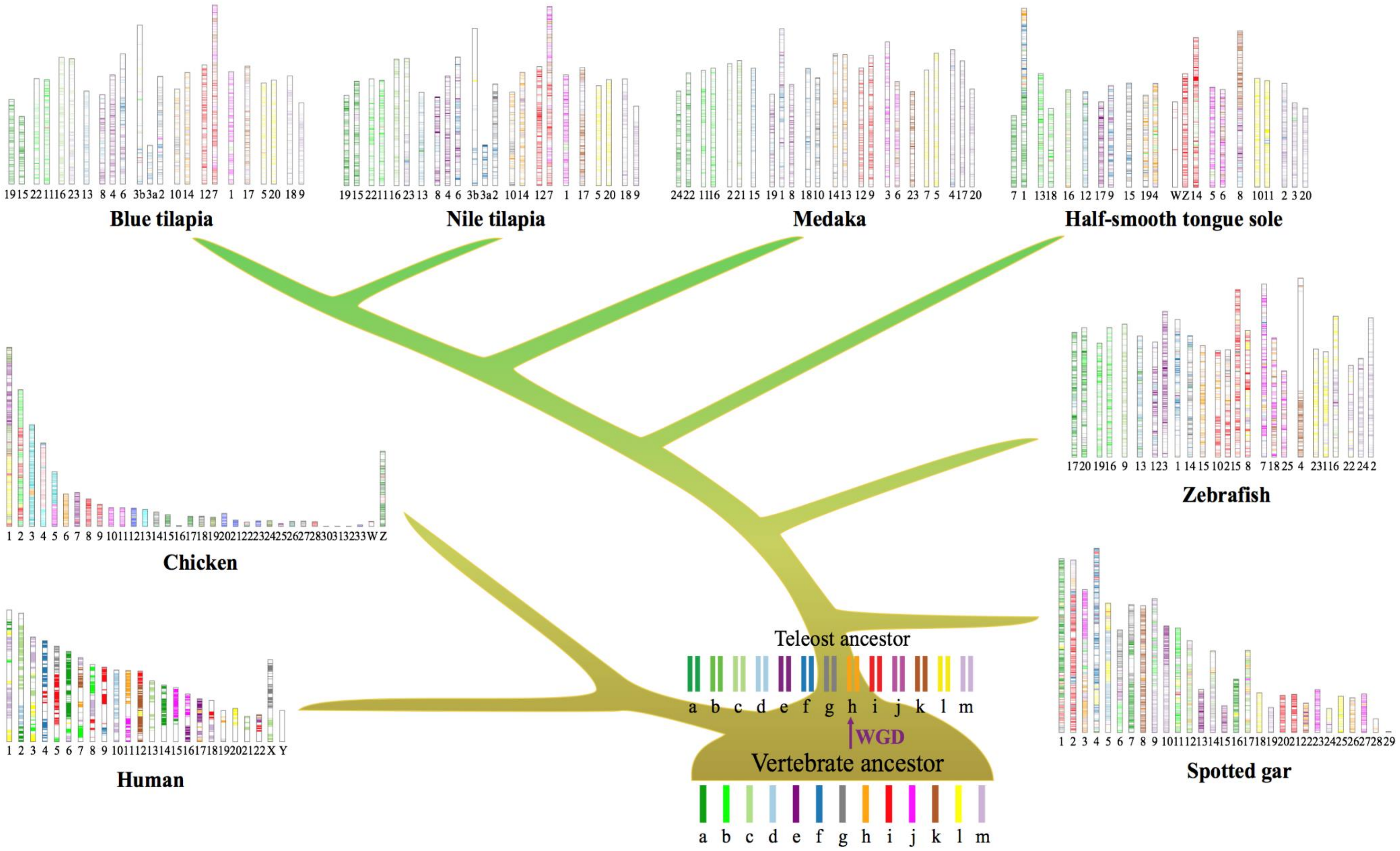


**Figure S1**. A schematic chart for the composition of ancestral chromosomes among various species. Vertebrate genomes evolved from the ancestral chromosomes through chromosomal loss, translocation, fission and fusion, and whole-genome duplication. The representative thirteen ancestral chromosomes are indicated with different colors.
