# Supplementary material for: Whole Genome Sequencing of the Blue Tilapia (Oreochromis aureus) Provides a Valuable Genetic Resource for Biomedical Research on Tilapias
Source: Mar Drugs. 2019 Jun 28;17(7):386. doi: 10.3390/md17070386 (PMC6669741; doi:10.3390/md17070386)
Supplement: Supplementary file 1 [file marinedrugs-17-00386-s001.zip › Supplementary Information/Table S1.docx]

**Table S1**. Statistics of the clean reads

| **Paired-end sequencing libraries** | **Insert Size** | **Raw Reads**  **(Gb)** | **Clean Reads (Gb)** | **Read Length (bp)** |
| --- | --- | --- | --- | --- |
| Illumina Reads | 250 bp | 74.55 | 68.87 | 125 |
|  | 500 bp | 24.86 | 23.28 | 125 |
|  | 800 bp | 19.74 | 18.68 | 125 |
|  | 2 kb | 31.99 | 19.61 | 125 |
|  | 5 kb | 33.82 | 15.64 | 125 |
|  | 10 kb | 27.24 | 7.52 | 125 |
|  | 20 kb | 27.69 | 7.93 | 125 |
| Total |  | 239.89 | 161.53 |  |
