# Supplementary material for: Whole Genome Sequencing of the Blue Tilapia (Oreochromis aureus) Provides a Valuable Genetic Resource for Biomedical Research on Tilapias
Source: Mar Drugs. 2019 Jun 28;17(7):386. doi: 10.3390/md17070386 (PMC6669741; doi:10.3390/md17070386)
Supplement: Supplementary file 1 [file marinedrugs-17-00386-s001.zip › Supplementary Information/Table S2.docx]

**Table S2**. Statistics of the blue tilapia genome assembly

| Parameter | Contig | | Scaffold | |
| --- | --- | --- | --- | --- |
|  | **Size (bp)** | **Number** | **Size (bp)** | **Number** |
| N90 | 6,374 | 21,593 | 78,053 | 1,418 |
| N80 | 14,440 | 13,022 | 238,365 | 746 |
| N70 | 24,595 | 8,624 | 481,702 | 482 |
| N60 | 37,652 | 5,895 | 774,198 | 332 |
| N50 | 53,192 | 4,040 | 1,095,619 | 231 |
| Longest | 651,854 | | 6,936,348 | |
| Total Size | 831,495,605 | | 924,787,968 | |
| Total(>100bp) Number(>100bp) | 106,865 | | 53,082 | |
| Total(>2kb) Number(>2kb) | 35,763 | | 5,561 | |
