# Supplementary material for: Whole Genome Sequencing of the Blue Tilapia (Oreochromis aureus) Provides a Valuable Genetic Resource for Biomedical Research on Tilapias
Source: Mar Drugs. 2019 Jun 28;17(7):386. doi: 10.3390/md17070386 (PMC6669741; doi:10.3390/md17070386)
Supplement: Supplementary file 1 [file marinedrugs-17-00386-s001.zip › Supplementary Information/Table S3.docx]

**Table S3**. Statistics of repeat sequences in blue and Nile tilapia genomes

| Repetitive element | | Nile tilapia | | | Blue tilapia | | |
| --- | --- | --- | --- | --- | --- | --- | --- |
| Order | Family | Number | Total (bp) | % | Number | Total (bp) | % |
| DNA | Sola | 7,007 | 1,536,337 | 0.15 | 18,326 | 2,255,704 | 0.24 |
|  | TcMar-Tc1 | 156,588 | 46,394,192 | 4.60 | 453,001 | 65,451,556 | 7.08 |
|  | hAT | 36,441 | 10,103,158 | 1.00 | 124,268 | 17,571,189 | 1.90 |
| LINE | L1 | 10,712 | 8,879,041 | 0.88 | 53,672 | 10,458,133 | 1.13 |
|  | L2 | 76,937 | 29,334,193 | 2.91 | 307,626 | 46,104,374 | 4.99 |
|  | Penelope | 28,509 | 7,214,522 | 0.71 | 98,380 | 14,354,972 | 1.55 |
|  | Rex-Babar | 38,996 | 19,208,630 | 1.90 | 128,621 | 20,845,930 | 2.25 |
| LTR | ERV1 | 10,756 | 6,450,995 | 0.64 | 18,782 | 2,923,225 | 0.32 |
|  | Gypsy | 29,201 | 13,615,743 | 1.35 | 109,985 | 19,878,940 | 2.15 |
| RC | Helitron | 3,882 | 2,685,111 | 0.27 | 15,270 | 1,707,432 | 0.18 |
| Unknown | Unknown | 350,007 | 97,456,302 | 9.66 | 35,413 | 11,391,887 | 1.23 |
| Satellite | Satellite | 10,061 | 7,662,597 | 0.76 | 1,428 | 671,220 | 0.07 |
|  | Simple | 322,732 | 15,543,664 | 1.54 | 27,802 | 5,436,791 | 0.59 |
