# Supplementary material for: Whole Genome Sequencing of the Blue Tilapia (Oreochromis aureus) Provides a Valuable Genetic Resource for Biomedical Research on Tilapias
Source: Mar Drugs. 2019 Jun 28;17(7):386. doi: 10.3390/md17070386 (PMC6669741; doi:10.3390/md17070386)
Supplement: Supplementary file 1 [file marinedrugs-17-00386-s001.zip › Supplementary Information/Table S4.docx]

**Table S4**. Gene annotation of the assembled blue tilapia genome

| **Gene Set** | | **Number** | **Average Transcript Length (bp)** | **Average CDS Length (bp)** | **Average Exons per Gene** | **Average Exon Length (bp)** | **Average Intron Length (bp)** |
| --- | --- | --- | --- | --- | --- | --- | --- |
| *De novo* | AUGUSTUS | 23,971 | 14,853 | 1,525 | 8.56 | 178.22 | 1,762 |
|  | Genescan | 40,180 | 14,774 | 1,449 | 7.94 | 182.55 | 1,919 |
| Homolog |  |  |  |  |  |  |  |
|  | Stickleback | 31,452 | 8,136 | 1,148 | 6.36 | 180.31 | 1,301 |
|  | Tilapia | 37,122 | 8,262 | 1,184 | 6.35 | 186.41 | 1,322 |
| Zebrafish | | 29,189 | 8,748 | 1,289 | 6.51 | 197.98 | 1,353 |
| Fugu | | 25,860 | 9,751 | 1,327 | 7.28 | 182.28 | 1,341 |
| Tetraodon | | 23,627 | 10,010 | 1,338 | 7.70 | 173.61 | 1,292 |
| Transcriptome |  | 34,894 | 11,801 | 2,766 | 9.24 | 299.27 | 1,095 |
| GLEAN | | 25,302 | 15,129 | 1,733 | 9.53 | 181.74 | 1,569 |
| Final gene set | | 23,117 | 15,586 | 1,789 | 9.90 | 180.71 | 1,549 |
