# Supplementary material for: Whole Genome Sequencing of the Blue Tilapia (Oreochromis aureus) Provides a Valuable Genetic Resource for Biomedical Research on Tilapias
Source: Mar Drugs. 2019 Jun 28;17(7):386. doi: 10.3390/md17070386 (PMC6669741; doi:10.3390/md17070386)
Supplement: Supplementary file 1 [file marinedrugs-17-00386-s001.zip › Supplementary Information/Table S5.docx]

**Table S5**. Functional assignments of the final gene set of the blue tilapia genome

| **Parameter** | **Gene Number** | **Percentage (%)** |
| --- | --- | --- |
| Total | 23,117 |  |
| InterPro | 19,480 | 84.26 |
| GO | 16,645 | 72.00 |
| KEGG | 17,629 | 76.26 |
| Swissprot | 21,294 | 92.11 |
| TrEMBL | 22,488 | 97.27 |
| Annotated | 22,573 | 97.65 |
| Unanotated | 544 | 2.35 |
